# RyhB in Avian Pathogenic *Escherichia coli* Regulates the Expression of Virulence-Related Genes and Contributes to Meningitis Development in a Mouse Model

**DOI:** 10.3390/ijms232415532

**Published:** 2022-12-08

**Authors:** Xia Meng, Yanfei Chen, Peili Wang, Mengping He, Yuxing Shi, Yuxin Lai, Guoqiang Zhu, Heng Wang

**Affiliations:** 1 Jiangsu Co-Innovation Center for the Prevention and Control of Important Animal Infectious Diseases and Zoonoses, College of Veterinary Medicine, Yangzhou University, Yangzhou 225009, China; 2 International Research Laboratory of Prevention and Control of Important Animal Infectious Diseases and Zoonotic Diseases of Jiangsu Higher Education Institutions, Yangzhou University, Yangzhou 225009, China; 3 Joint International Research Laboratory of Agriculture and Agri-Product Safety, The Ministry of Education of China, Yangzhou 225009, China

**Keywords:** RyhB, virulence regulation, APEC, meningitis development

## Abstract

Avian pathogenic *Escherichia coli* (APEC) is an important member of extraintestinal pathogenic *Escherichia coli* (ExPEC). It shares similar pathogenic strategies with neonatal meningitis *E. coli* (NMEC) and may threaten human health due to its potential zoonosis. RyhB is a small non-coding RNA that regulates iron homeostasis in *E. coli*. However, it is unclear whether RyhB regulates meningitis occurrence. To investigate the function of RyhB in the development of meningitis, we constructed the deletion mutant APEC XM∆*ryhB* and the complemented mutant APEC XM∆*ryhB*/p*ryhB*, established a mouse meningitis model and evaluated the role of RyhB in virulence of APEC. The results showed that the deletion of *ryhB* decreased biofilm formation, adhesion to the brain microvascular endothelial cell line bEnd.3 and serum resistance. RNA-seq data showed that the expression of multiple virulence-related genes changed in the *ryhB* deletion mutant in the presence of duck serum. Deletion of *ryhB* reduced the clinical symptoms of mice, such as opisthotonus, diarrhea and neurological signs, when challenged with APEC. Compared with the mice infected with the wild-type APEC, fewer histopathological lesions were observed in the brain of mice infected with the *ryhB* deletion mutant APEC XM∆*ryhB*. The bacterial loads in the tissues and the relative expression of cytokines (*IL-1β*, *IL-6*, and *TNF-α*) in the brain significantly decreased when challenged with the APEC XM∆*ryhB*. The expressions of tight junction proteins (claudin-5, occludin and ZO-1) were not reduced in the brain of mice infected with APEC XM∆*ryhB*; that is, the blood-brain barrier permeability of mice was not significantly damaged. In conclusion, RyhB contributes to the pathogenicity of APEC XM in the meningitis-causing process by promoting biofilm formation, adhesion to endothelial cells, serum resistance and virulence-related genes expression.

## 1. Introduction

Avian pathogenic *Escherichia coli* (APEC) is an important member of the extraintestinal pathogenic *Escherichia coli* (ExPEC) subgroup. It causes severe systemic disease, such as bacteremia and meningitis in poultry and mouse models [1,2]. APEC and ExPEC strains have a close relationship and share some common genotypic characteristics and virulence factors to cause disease [2,3,4]. The genome sequence of APEC has shown strong similarity with human ExPEC [5]. APEC serotype O18 strains utilize similar pathogenic mechanisms to those of neonatal meningitis *E. coli* (NMEC) strains to cause meningitis [6]. This evidence shows that APEC may be a potential zoonotic bacterium. Bacterial meningitis is a serious disease with limited improvement in mortality and morbidity. The pathogenesis of meningitis is not completely understood [7,8]. APEC XM (O2:K1), the strain used in this study, is a B2 phylogenetic group ExPEC. This strain was isolated from the brain of ducks with serious sepsis and meningitis. Many virulence factors such as type VI secretion systems, the Rcs regulatory system, the global regulator ferric uptake regulator (Fur) and outer membrane proteins OmpF and OmpC have been identified in this strain [9,10,11]. However, the pathogenic mechanisms, especially the meningitis-causing mechanism of APEC, have not been clarified. In addition, studies on the pathogenesis of meningitis have mainly focused on virulence proteins and protein regulators, while the role of RNA in meningitis development has been rarely reported in *E. coli* k1 [12] and has not been reported in APEC.

Small non-coding RNAs (sRNAs) are a type of RNA that regulate the expression of their target genes in response to environmental changes [13]. Many sRNAs play a major role in global gene regulatory networks and always modulate the gene expression of numerous targets at a post-transcriptional level by base pairing with mRNA targets [14,15,16]. RyhB is a trans-encoded sRNA that was first identified in *E. coli* in 2001 [17]. Since then, RyhB has been found and studied in a variety of bacteria, such as *Salmonella* [18,19], *Vibrio cholerae* [20], *Yersinia pestis* [21], *Klebsiella pneumoniae* [22] and *Shigella dysenteriae* [23]. RyhB, repressed by Fur, is crucial for iron homeostasis by modulating more than 50 genes involved in iron uptake and utilization [24,25,26]. In addition to iron metabolism regulation, RyhB regulates diverse physiological processes of bacteria, including nitrate homeostasis [27], acid resistance [28], adaption to oxidative stress [29] and motility [30]. In some pathogens, RyhB is involved in a variety of virulence-related phenotypic characteristics, such as biofilm formation [20], survival within eukaryotic epithelial cells and macrophages [19,31] and the expression of virulent genes [32]. However, the virulence regulation function of RyhB in APEC has not been clarified, and the regulation of meningitis development by RyhB has not been reported. To study the role of RyhB in meningitis development and how RyhB regulates meningitis development, we deleted the *ryhB* gene in APEC XM and evaluated a series of phenotypes associated with meningitis development in the *ryhB* deletion mutant in vitro and in a mouse meningitis model.

## 2. Results

### 2.1. RyhB Is Induced by Duck Serum and Contributes to the Survival of APEC in Duck Serum

**Figure 1:**
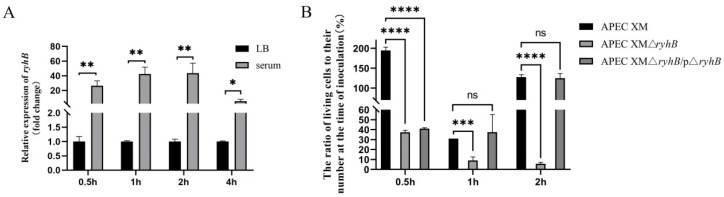
Determination of *ryhB* expression and survival of APEC in serum. (**A**) The relative expression of *ryhB* was determined using quantitative real-time PCR (qRT-PCR). Four time points, 0.5 h, 1 h, 2 h and 4 h, were selected for incubating APEC XM in LB medium and duck serum. The relative expression of *ryhB* of APEC XM that grew in LB medium for 0.5 h, 1 h, 2 h and 4 h was defined as 1. (**B**) The survival rates of APEC in duck serum. The survival rate at each time point was determined by calculating the ratio of the number of bacteria at 0 h to the number of bacteria at different serum incubation times for each strain. The data were analyzed with one-way ANOVA and are expressed as the mean ± standard error of the mean for three independent experiments. (****, *p* < 0.0001; ***, *p* < 0.001; **, *p* <0.01; *, *p* < 0.05; ns: not significant, *p* > 0.05).

To investigate the response of RyhB to duck serum, the transcriptional levels of *ryhB* at different time points were detected using quantitative real-time PCR (qRT-PCR). The results showed that the expression of *ryhB* changed little when the APEC XM was grown in LB medium from 0.5 h to 4 h. The expression of *ryhB* was significantly induced by more than 20-fold when grown in duck serum, compared to that in LB medium. The most distinct induction was more than 40-fold in duck serum at 2 h (Figure 1A). These findings indicated that the serum induced the expression of *ryhB*. The survival assay of the wild-type (WT) strain APEC XM, the *ryhB* deletion mutant APEC XMΔ*ryhB* and the complemented strain APEC XMΔ*ryhB*/p*ryhB* in duck serum showed that the survival rate of APEC XMΔ*ryhB* increasingly declined, while that of APEC XM first decreased and then increased from 0.5 h to 2 h. The survival of APEC XMΔ*ryhB*/p*ryhB* was restored (Figure 1B). This indicated that the deletion of *ryhB* led to the decrease in the survival of APEC. In other words, RyhB contributed to the survival and multiplication of APEC XM in the serum.

### 2.2. RyhB Contributes to Adhesion to bEnd.3 Cells

**Figure 2:**
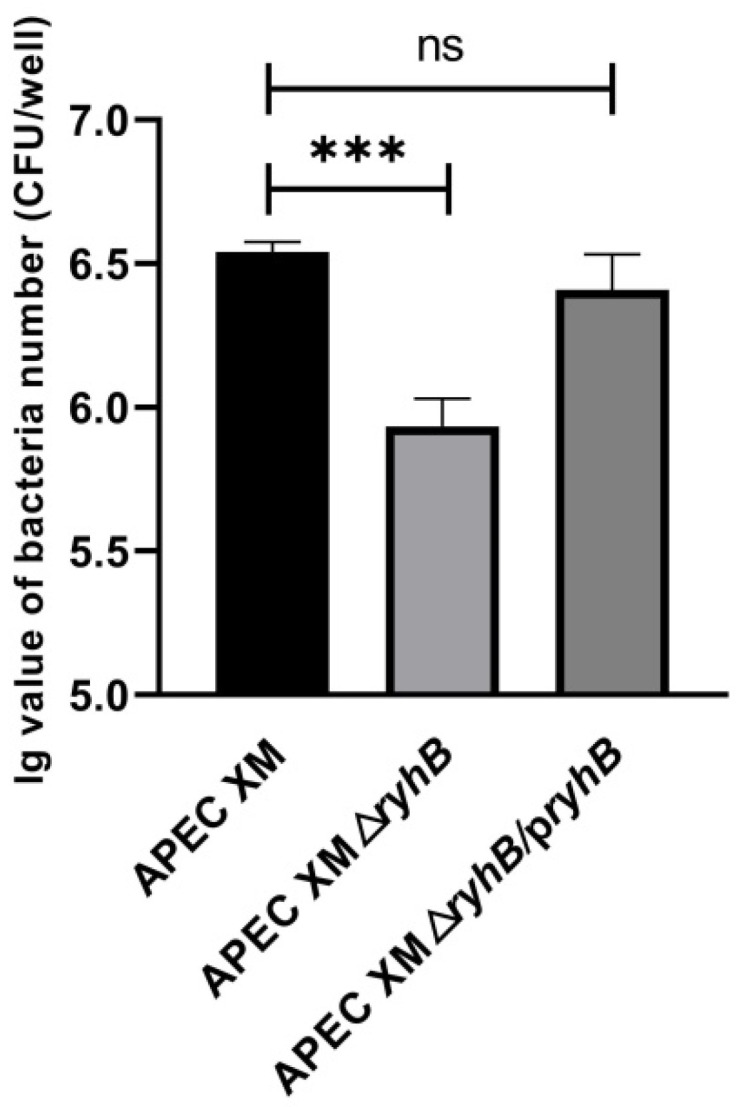
Adherence to bEnd.3 cells by APEC XM, APEC XMΔ*ryhB* and APEC XMΔ*ryhB*/p*ryhB*. The data were analyzed with one-way ANOVA and are expressed as the mean ± standard error of the mean for three independent experiments. (***, *p* < 0.001; ns, *p* > 0.05).

Adhesion of brain microvascular endothelial cells is an important step for meningitis. The adhesion abilities of APEC XM, APEC XMΔ*ryhB* and APEC XMΔ*ryhB*/p*ryhB* were determined using the mouse brain microvascular endothelial cell line bEnd.3. Compared with APEC XM, the adhesion of APEC XMΔ*ryhB* significantly decreased. The APEC XMΔ*ryhB*/p*ryhB* almost completely restored the adhesion ability (Figure 2). This indicated that RyhB was responsible for adhesion to bEnd.3 cells.

### 2.3. RyhB Contributes to the Biofilm Formation of APEC XM

**Figure 3:**
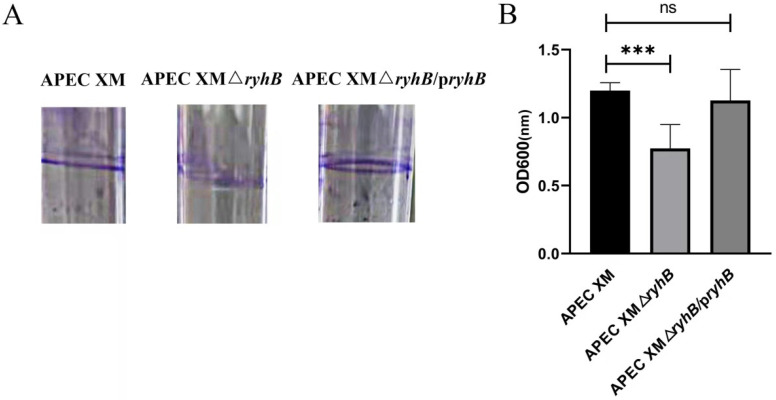
Determination of biofilm formation. (**A**) Qualitative assay of biofilm formation in the test tube. (**B**) Quantitative assay of biofilm formation in 96-well microtiter plates. The OD_600_ absorbance was measured to quantify the biofilm formation. All assays were performed in triplicate. Error bars represent the standard errors of the means. The comparison of biofilm formation between mutants and APEC XM was statistically analyzed using one-way ANOVA (***, *p* < 0.001; ns, *p* > 0.05).

The biofilm formation was qualitatively and quantitatively determined. In the qualitative assay, a crystal violet ring at the interface between the air and the liquid medium was observed. The crystal violet-stained biofilm formed by APEC XMΔ*ryhB* was less obvious than that formed by APEC XM (Figure 3A). The quantitative assay showed that the biofilm formation ability of APEC XMΔ*ryhB* significantly decreased (Figure 3B). The biofilm formation in APEC XMΔ*ryhB*/p*ryhB* was partly restored. Thus, RyhB appears to be important in the biofilm formation of APEC.

### 2.4. RyhB Affects the Expression of Multiple Virulence-Related Genes When Growing in Duck Serum

**Figure 4:**
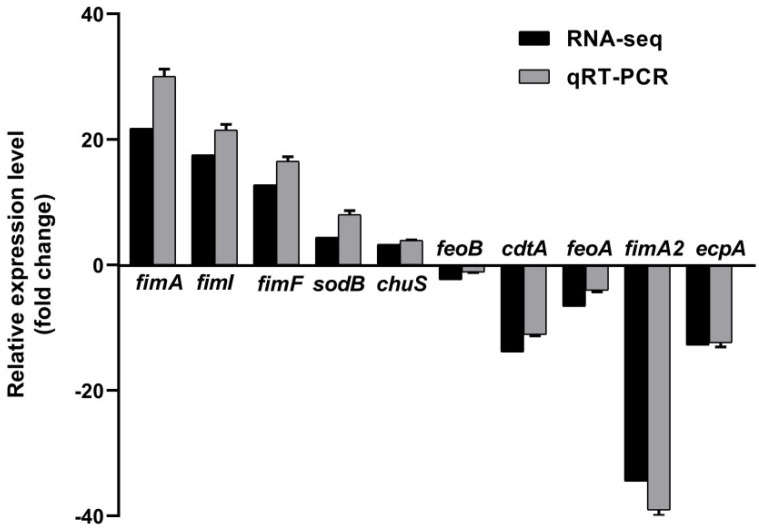
Verification of randomly selected differential expression of genes (DEGs) screened from RNA-seq data by qRT-PCR.

The survival of APEC in blood is very important for the occurrence of bacteremia, which is necessary for meningitis development. To study the role of RyhB in serum resistance, we compared the transcriptional level of whole genes in APEC XM with that in APEC XMΔ*ryhB* by RNA-seq technology. After a series of quality assessment and sequencing, the clean reads with high quality were obtained and mapped onto the published reference genome (*E. coli* strain ExPEC XM, NCBI accession No. CP025328). The Pearson correlation of all samples displayed high reproducibility within groups (three biological replicates in each group). The analysis results of the differential expression of genes (DEGs) showed that 283 DEGs were identified between the APEC XM group and APEC XMΔ*ryhB* group; i.e., 147 up-regulated DEGs and 136 down-regulated DEGs. To validate the accuracy of the DEG screening result, five up-regulated DEGs (*fimA*, *fimF*, *fimI*, *sodB* and *chuS*) and five down-regulated DEGs (*neuB*, *feoB*, *cdtA*, *feoA* and *fimA2*) (details in Supplementary Table S1) were randomly selected and their expressions were detected using qRT-PCR. The result showed that the relative quantity (RQ) values of these genes were consistent with the data obtained from RNA-seq (Figure 4). This indicated that the RNA-seq data were reliable and accurate.

**Figure 5:**
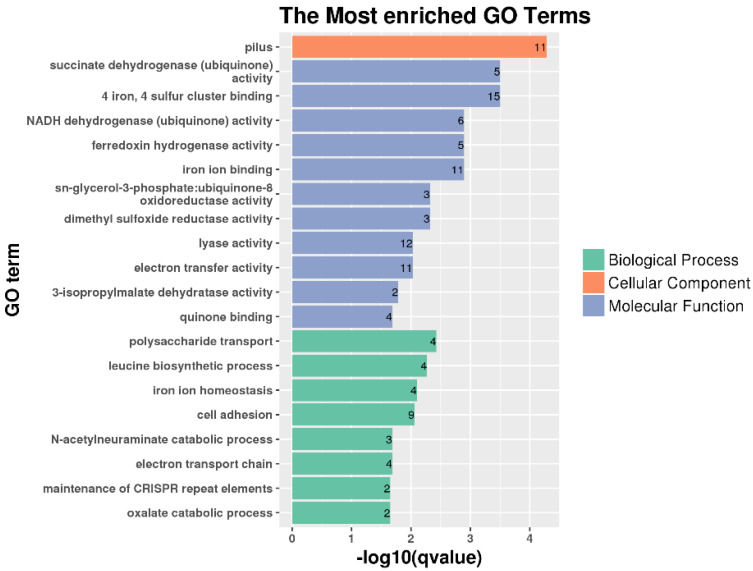
The top 20 enriched Gene Ontology (GO) terms that identified from the APEC XMΔ*ryhB* compared to the wild-type (WT) strain APEC XM.

Functional annotation of DEGs was performed using Gene Ontology (GO) enrichment analysis and Kyoto Encyclopedia of Genes and Genomes (KEGG) databases. The 20 most enriched GO terms are shown in Figure 5. Eleven fimbrial genes, including *fimA*, *fimH*, *fimF* and *ecpD*, were enriched in the “pilus” term in the “cellular component” part. RyhB is an important regulator of iron homeostasis [26]. The GO enrichment analysis showed that multiple iron metabolism-related DEGs were enriched in the “molecular function” part, including terms “4 iron, 4 sulfur cluster binding”, “ferredoxin hydrogenase activity” and “iron ion binding”, as well as the term “iron ion homeostasis” in the “biological process” part. In addition, “cell adhesion”, which is important for the pathogenicity of *E. coli*, was also enriched. This indicated that RyhB affected iron metabolism and the virulence of APEC XM when challenged with duck serum.

**Figure 6:**
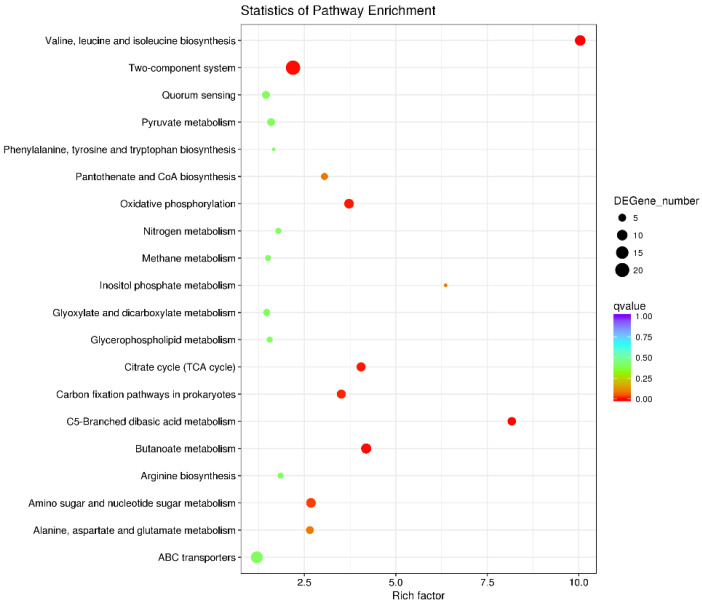
Enrichment analysis of KEGG pathways for DEGs. The top 20 enriched KEGG pathways were displayed. The size of each circle represents DEGs number in each pathway. The colour represents the corrected *q* value of each pathway.

The top 20 enriched KEGG pathways are shown in Figure 6. In addition to pathways related to substance metabolism, such as pyruvate metabolism, nitrogen metabolism, TCA cycle and butanoate metabolism, virulence-related pathways have attracted attention. Two-component systems are signal transduction regulatory circuits that respond to environmental stimuli. They are also involved in the regulation of virulence [33]. Twenty-one DEGs were enriched in the two-component system pathway, nine of which (such as formate dehydrogenase-N subunit alpha gene *fdnG*, and flagellar transcriptional regulator genes *flhC* and *flhD*) showed increased expression in the APEC XMΔ*ryhB*. This indicated that RyhB inhibited the expression of the abovementioned nine DEGs. Another 12 genes, including ferric enterobactin receptor CXG97_RS26865 and acid-sensing system genes *evgA* and *evgS*, showed decreased expression in the APEC XMΔ*ryhB*. Quorum sensing is a cell-cell communication system in bacteria. It modulates virulence, including biofilm formation, the secretion of toxins and the production of bacteriocins, and is important for the pathogenesis of bacteria [34]. Our RNA-seq data showed that five DEGs, i.e., *trpE* (anthranilate synthase component), *flhC* and *flhD* (Flagellar transcriptional regulator), *rcsA* (transcriptional regulator) and *ygjI* (amino acid permease), were enriched in the quorum sensing pathway. The expressions of these five genes increased in the APEC XMΔ*ryhB*.

In addition to the above DEGs enriched in GO and KEGG analysis, other virulence-related DEGs were also of interest. The serum-resistance-related genes *neuD*, *neuC*, *neuB*, the capsule biosynthesis genes *kpsF*, *kpsC*, *kpsS*, *kpsE* and *kpsD*, and the lipopolysaccharide (LPS) biosynthesis genes *arnB* and *arnC* were down-regulated in the APEC XMΔ*ryhB* mutant. The transcriptional levels of biofilm formation regulator gene *ybaJ* and inner membrane protein encoding gene *pgaD*, that is involved in biofilm formation, were down-regulated by more than two-fold in the APEC XMΔ*ryhB* mutant. Type I fimbria encoded genes *fimH*, *fimA2*, *fimF*, *fimC* and *fimG* were up-regulated, while *fimA* was down-regulated in the APEC XMΔ*ryhB* mutant. All of the abovementioned DEGs are summarized in Table S2.

### 2.5. RyhB Is Required for the Pathogenicity of APEC XM to ICR Mice

The role of RyhB in the pathogenicity of APEC XM was evaluated by infecting an Institute of Cancer Research (ICR ) mouse model with APEC XM, APEC XMΔ*ryhB* and APEC XMΔ*ryhB*/p*ryhB*. The clinical signs appeared 6 h after infection and were evident from 10 to 12 h after infection in the APEC XM infected mice and the APEC XMΔ*ryhB*/p*ryhB* infected mice. Most mice showed symptoms such as depression, eyelid closure with thick red eye discharge, diarrhea and neurological symptoms (clinical score 2–3) (Figure S1). The mice in the APEC XMΔ*ryhB* infection group did not present apparent symptoms (clinical score 0). This result indicated that the deletion of *ryhB* attenuated the virulence of APEC XM to mice.

**Figure 7:**
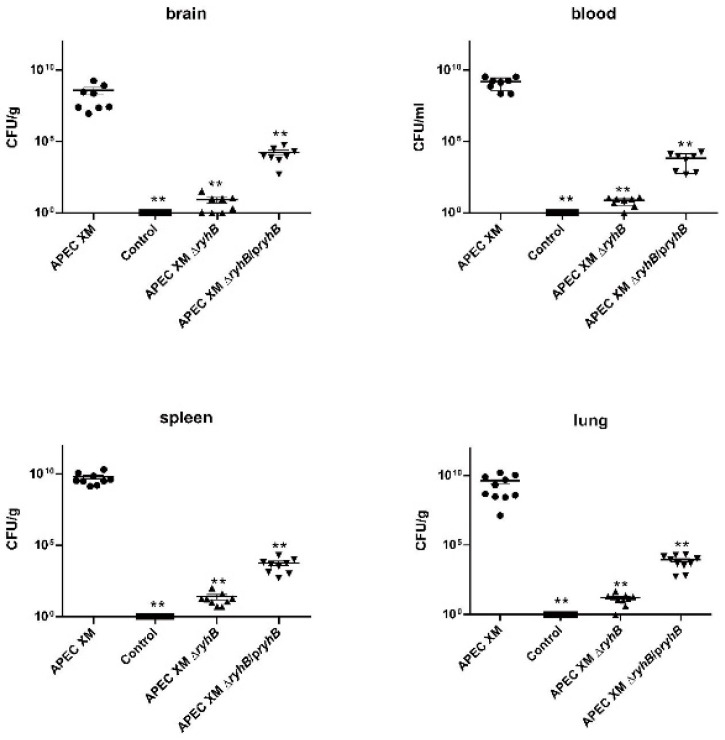
Bacterial loads in the brain, blood, spleen and lungs of infected mice after 12-h infection. Data are expressed as the mean ± standard deviation of triplicate experiments. Significant differences between the APEC XM and APEC XMΔ*ryhB* are indicated (**, *p* < 0.01).

The bacterial loads in the brain, spleen and lungs of mice were significantly decreased in the APEC XMΔ*ryhB* infection group, compared with those in the APEC XM infection group (Figure 7). This indicated that the deletion of *ryhB* decreased the colonization of APEC XM in the tissues of mice. The complementation of *ryhB* partly restored the colonization ability of the APEC XM.

### 2.6. RyhB Contributes to Brain Lesions of Mice

**Figure 8:**
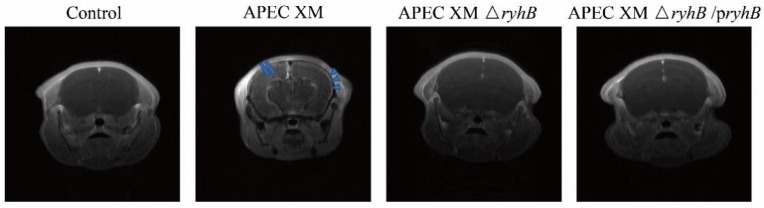
Lesions of mouse brains examined with MRI scanning. Abnormal contrast continuous linear enhancement of the pia mater (blue arrow) and diffusion enhancement of the cerebral parenchyma (blue arrowhead) are shown in the mouse infected with APEC XM.

**Figure 9:**
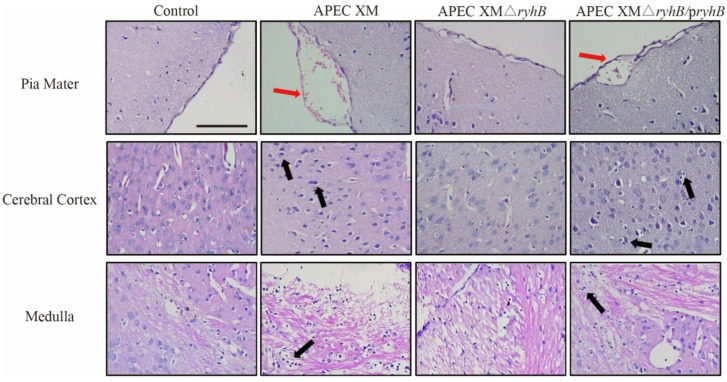
Histopathological analysis of the brain tissue of mice infected with APEC XM, APEC XMΔ*ryhB* and APEC XMΔ*ryhB*/p*ryhB*, and non-infected mice. Sections were stained with hematoxylin-eosin and visualized under optical microscopy (bar = 100 µm). The thickened pia mater with hemorrhage (red arrowheads) was observed in the brain section of mice infected with APEC XM and APEC XMΔ*ryhB*/p*ryhB*. Leukocyte infiltration (black arrowheads) was observed in the cerebral cortex of mice infected with APEC XM and APEC XMΔ*ryhB*/p*ryhB*.

The brain lesions of infected mice were evaluated using an MRI scan and histological analysis. The enhanced T1-weighted imaging (T1WI) showed an abnormal contrast continuous linear enhancement of the pia mater, widened sulci and a diffusion enhancement of the cerebral parenchyma in the brain in mice challenged with APEC XM. Compared with the APEC XM infection group, the mice challenged with APEC XMΔ*ryhB* did not display abnormal MRI features in the brain (Figure 8). The histopathological analysis showed leukocyte infiltration in the cerebral cortex, and thickening and hemorrhages in the pia mater of mice infected with APEC XM. The meninge was discontinuous and detached from the cerebral cortex. There were no obvious lesions in the mice of the APEC XMΔ*ryhB* infection group compared with those in the APEC XM infection group (Figure 9). The brains of mice in the APEC XMΔ*ryhB*/p*ryhB* infection group presented similar histopathological characteristics as the mice infected with APEC XM. These results indicated that RyhB contributed to brain lesions of mice.

### 2.7. RyhB Helped APEC XM to Damage the Integrity of the Blood–Brain Barrier

**Figure 10:**
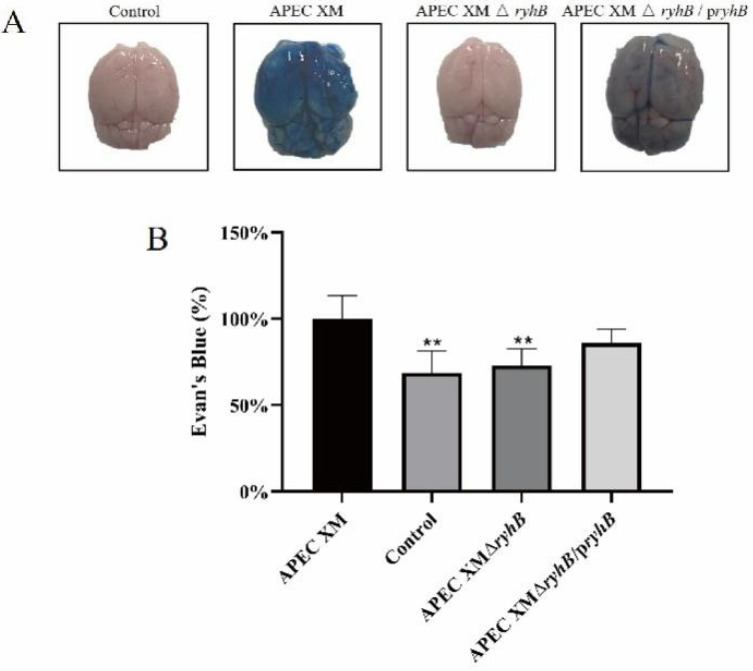
The integrity determination of blood-brain barrier by EB staining. (**A**) The dorsal view of brains after EB injection. (**B**) Quantification of EB that penetrated the brain. The amount of EB was calculated by measuring the absorbance at 630 nm. The data of the APEC XM group were set as 100%. Data were analyzed with SPSS 17.0 software using one-way ANOVA. Error bars represent the standard errors of the means. Significant differences between the APEC XM and APEC XMΔ*ryhB* are indicated (**, *p* < 0.01).

The permeability of Evans blue (EB) in the brain can be used to evaluate the integrity and permeability of the blood-brain barrier (BBB). The amount of EB that penetrated the brain was measured in the mice infected with APEC XM, APEC XMΔ*ryhB* and APEC XMΔ*ryhB*/p*ryhB*. The results showed that amounts of EB stain accumulated in the brain of mice infected with APEC XM. The content of EB in APEC XMΔ*ryhB* was significantly decreased compared with that in the APEC XM infection group, but partly restored in the APEC XMΔ*ryhB*/p*ryhB* infection group. (Figure 10). This indicated that RyhB helped APEC XM to damage the integrity of the BBB.

### 2.8. RyhB Affects the Expressions of IL-1β, IL-6 and TNF-α

**Figure 11:**
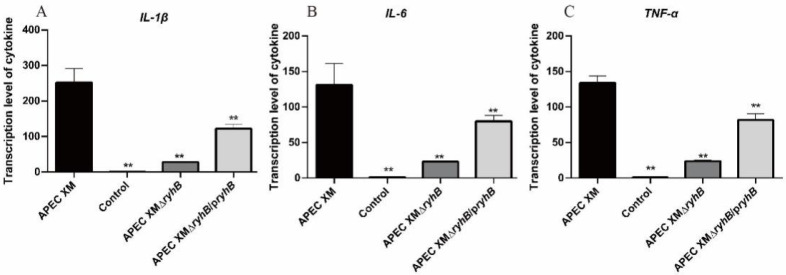
The mRNA transcript levels of the inflammatory cytokines *IL-1β* (**A**), *IL-6* (**B**) and *TNF-α* (**C**) were determined using qRT-PCR. *GAPDH* was used as the normalizing internal standard. The results were analyzed with one-way ANOVA and are presented as the means ± standard errors of the mean for three independent experiments. Significant differences between the APEC XM and other groups are indicated (**, *p* < 0.01).

The relative expressions of *IL-1β*, *IL-6* and *TNF-α* mRNA were measured in the brain tissues using qRT-PCR. Compared to the expression in the control group, the relative expressions of *IL-1β*, *IL-6* and *TNF-α* were significantly increased in the brains of mice challenged with APEC XM. The expressions of these three inflammatory cytokines were decreased in the APEC XMΔ*ryhB* infection group, compared with the APEC XM infection group, while they were partly restored in the APEC XMΔ*ryhB*/p*ryhB* infection group (Figure 11). It can be concluded that the deletion of *ryhB* reduced inflammation caused by APEC XM in mice.

### 2.9. RyhB Affects the Expression of Tight Junction Proteins

**Figure 12:**
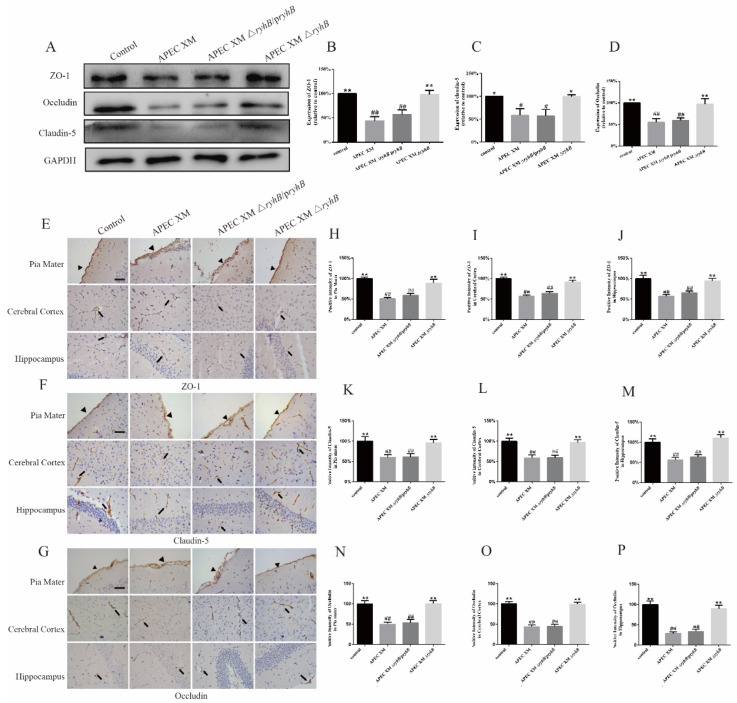
The expressions of ZO-1, claudin-5 and occludin in the brain tissues were measured using Western blot and immunohistochemistry. (**A**–**D**) The expressions of ZO-1 (**A**,**B**), claudin-5 (**A**,**C**) and occludin (**A**,**D**) in the brain were measured using Western blot. GAPDH was used as a loading control. The protein expression in control group is defined as 100%. (**E**–**P**) The expressions of ZO-1, claudin-5 and occludin in the brain were measured using immunohistochemistry. The representative immunohistochemistry staining of ZO-1 (**E**), claudin-5 (**F**) and occludin(**G**) proteins were displayed in the brain sections of infected mice (bar = 20 µm). The black arrowheads point to pia maters and the black arrows point to microvascular. The percentage of positive staining intensity of ZO-1 (**H**–**J**), claudin-5 (**K**–**M**) and occludin (**N**–**P**) in pia mater, cerebral and hippocampus were calculated by defining the relative optical density of the control group as 100%, and the relative optical densities of other groups are normalized by the control group. These results are presented as the means ± standard error of the mean for three independent experiments. (**, *p* < 0.01, *, 0.01 < *p* < 0.05, versus APEC XM group; ##, *p* < 0.01, #, 0.01 < *p* < 0.05 versus control group).

The expressions of tight junction proteins ZO-1, occludin and claudin-5 were measured using Western blot and immunohistochemical staining in vivo. Compared with the control group, the expressions of ZO-1, occludin and claudin-5 were significantly lower in the brains of mice challenged with APEC XM and APEC XMΔ*ryhB*/p*ryhB*. The expressions in the APEC XMΔ*ryhB* infection group were similar to those in the control group (Figure 12A–D). The immunohistochemical staining of ZO-1, occludin and claudin-5 (Figure 12E–G) in the pia mater, cerebral cortex and hippocampus of the mice brains showed that, compared with the negative control group, the expressions of ZO-1 (Figure 12H–J), claudin-5 (Figure 12K–M) and occludin (Figure 12N–P) were significantly decreased in the mice brains infected with APEC XM and APEC XMΔ*ryhB*/p*ryhB*. The expressions in the APEC XMΔ*ryhB* infection group were similar to those in the control group. These results indicated that RyhB contributes to the disruption of the integrity and permeability of the BBB.

## 3. Discussion

*E. coli* meningitis is an important cause of mortality and morbidity worldwide and usually leads to neurological sequelae in survivors [35]. Cases of meningitis are increasingly occurring in poultry and are causing economic losses to the poultry industry. Investigation of the pathogenesis of meningitis is important for the prevention and therapy of *E. coli* meningitis. Previous studies on the pathogenesis of meningitis have mainly focused on virulence proteins. In recent years, the function of sRNAs in pathogen virulence regulation has been of interest [15,36]. In our study, the role of sRNA RyhB in meningitis development was investigated.

In the process of meningitis occurrence, bacteria colonize in mucosa, invade, survive and multiply in the bloodstream, and then cause high levels of bacteremia. After that, bacteria cross the BBB, and invade the meninges and central nervous system. Subsequently, bacteria increase the permeability of the BBB, promoting the release of proinflammatory compounds and ultimately leading to neuronal injury [8]. In this pathogenic process, adhesion and invasion to cells and bacteremia are important factors. Therefore, we first studied the role of RyhB in serum resistance, adhesion to cells and biofilm formation of APEC in vitro, and analyzed the transcriptional levels of the whole mRNA when APEC encountered duck serum.

In the early stage of infection, ExPEC evades or inhibits the host immune system and proliferates in the bloodstream, which leads to bacteremia. High levels of bacteremia are considered to be a necessary but insufficient condition for the occurrence of *E. coli* meningitis. The resistance of bacteria to serum is very important for the development of bacteremia [10]. Bacteria without serum resistance usually do not have the ability to infect the organs of the host [37]. Therefore, it is important to study the function of RyhB in serum resistance.

As an sRNA, RyhB is induced by environmental signals to response to stress, such as iron-limited conditions [38,39], anaerobic conditions [40] and survival in macrophages [41]. Serum contains a variety of bactericidal factors; it is also a stressful environment for APEC. To mimic natural host infection, we detected the expression of *ryhB* when APEC was incubated with duck serum. The results showed that RyhB was significantly induced when APEC was cultured in duck serum, compared with that cultured in LB medium. This indicated that RyhB responded to bactericidal factors in the serum and helped APEC to adapt to this stressful environment. Survival rate determination showed that the survival rate of APEC markedly decreased due to the deletion of *ryhB*, especially when incubated for 2 h. This means that RyhB is critical for serum resistance. As an RNA regulator, RyhB controls a variety of physiological processes of bacteria including iron homeostasis by regulating the expression of multiple target genes. To identify the target genes of RyhB responsible for serum resistance, the transcriptomes of APEC XM and APEC XMΔ*ryhB* grown in duck serum were compared. The RNA-seq data showed that 283 DEGs were screened out. Cell components K-Capsules [10,42], LPS [43] and outer membrane protein OmpA play an important role in serum resistance in *E. coli* [44]. The expression of many serum-resistance-related genes including *neuD*, *neuC*, *neuB*, Capsules biosynthesis genes *kpsF*, *kpsC*, *kpsS*, *kpsE* and *kpsD*, and LPS biosynthesis genes *arnB*, *arnA* significantly decreased in the *ryhB* deletion mutant. We inferred that serum resistance of APEC is associated with the upregulation of capsules and LPS synthesis genes, and other serum survival-related genes that are regulated by RyhB. In this sense, RyhB indirectly affects serum resistance. Another example of indirect effects on serum resistance is the cold shock proteins (CSPs) CspC and CspE. Both of them play an important role in serum resistance of Septicemic *E. coli* [45]. The stability of many transcripts. including serum survival genes *tdcA* and *clpX*, were controlled by CspC and CspE. Interestingly, the transcription of RyhB and Fur were related to both CspC and CspE [45]. Fur is also a major regulator of ExPEC response to serum [46]. It is assumed that there is a link between CSPs, RyhB and Fur in affecting serum resistance.

*E. coli* meningitis follows a high-degree of bacteremia and invasion of BBB. Adhesion and invasion to the BBB are essential steps in the development of *E. coli* meningitis. Brain microvascular endothelial cells (BMECs) are an important component of the BBB. *E. coli* adhere to BMECs only in the case of bacteremia [47]. Our study showed that the deletion of *ryhB* significantly attenuated the adhesion of APEC XM to bEnd.3 cells. This indicated that RyhB contributed to the adhesion ability of APEC to host cells. It is assumed that RyhB up-regulates the expression of adhesion-related genes. There are many virulence factors involved in adhesion which contain fimbriae adhesin and non-fimbriae adhesin [48,49]. Type I fimbriae, a hairlike protuberance of *E. coli*, have been confirmed to be involved in the adhesion to eukaryotic cells and biofilm formation [50]. In F18ab enterotoxigenic *E. coli*, flagella can promote the adhesion and invasion of bacteria to HeLa cells and to piglet intestinal epithelial cells [51]. Our RNA-seq data revealed that RyhB promoted the expression of *fimA* but repressed the expression of other type I fimbriae genes including *fimH*, *fimA2*, *fimF*, *fimC* and *fimG*. Moreover, RyhB repressed the expression of flagellar transcriptional activator encoded genes *flhC* and *flhD*. This result does not seem to be consistent with the result of the adhesion test to bEnd.3 cells. We speculate that there may be many factors involved in adhesion, and that changes in the expression of a few genes do not reflect overall adhesion characteristics. Adhesion is a comprehensive effect of various adhesion factors. Moreover, transcriptome data are derived from conditions of resistance to serum rather than adhesion of cells, and RyhB is highly influenced by the environment. The regulatory role of RyhB on target genes may also be different under different environmental conditions. However, regardless of how RyhB regulates the expression of adhesion-related genes, what we confirm is that RyhB contributes to the adhesion of APEC to bEnd.3 cells.

Biofilms are responsible for most chronic and recurrent infections [52]. Biofilm formation renders bacteria less susceptible to harsh environmental conditions, such as antibiotics and the host’s immune system. A previous study showed that RyhB in *Vibrio cholera* affected biofilm formation [20]. sRNA rss04 positively regulates the biofilm formation of *Streptococcus suis*. In the mouse meningitis model, the biofilm state in the brain of mouse contributed to *S. suis* virulence [53]. Based on the biofilm formation assay in vitro, we found that the deletion of *ryhB* decreased the biofilm production of APEC. This indicates that RyhB contributes to biofilm formation in APEC. It is assumed that RyhB regulates the virulence of APEC by affecting biofilm formation.

APEC can infect poultry and cause bacteremia and meningitis in a newborn SD rat model and a 4-week-old mouse model [2,54]. Therefore, the mouse model is suitable for virulence tests of APEC. We constructed a mouse model to study the function of RyhB in the process of meningitis development in mice infected with APEC. The clinical signs, bacterial loads in tissues, brain lesion and BBB damage were evaluated in the infected ICR mice. A high degree of bacteremia is necessary for *E. coli* meningitis. Our study showed that the bacterial load in blood was significantly decreased in the *ryhB* deletion mutant. Combined with the result of serum resistance assay, we assumed that the deletion of *ryhB* leads to a decreased survival of APEC in the blood, resulting in fewer bacteria in the blood. The low level of the bacterial population may be not enough to cause meningitis. The results of MRI and histopathological analysis showed that APEC XM damaged the meninges and caused lesions in the brain. The deletion of *ryhB* decreased the ability of APEC to damage the mice brains. BBB is one of the tightest barriers in the body that protects the brain from bacteria and toxins in the blood [55]. During meningitis, the permeability of BBB and the expression of cytokines *IL-1β*, *IL-6* and *TNF-α* increased. Tight junctions (TJs) were disrupted [56,57]. We demonstrated that APEC XM infection led to an increase in the expression of *IL-1β*, *IL-6* and *TNF-α*. The expressions of tight junction proteins ZO-1, claudin-5 and occludin in brains infected with APEC XM decreased compared with the mice in the control group. The deletion of *ryhB* weakened the damage of the permeability of BBB, reduced the inflammation and disruption of TJs. This indicated that RyhB is an important factor for meningitis occurrence.

Although we have revealed the critical role of RyhB in the process of meningitis development caused by APEC, the mechanism of RyhB regulating the development of meningitis has not been clarified. Whether RyhB directly disrupts the BBB and causes brain damage in mice is unclear. Based on the in vitro and in vivo tests in our study, we speculate that RyhB-mediated survival in blood is crucial for bacteremia. The deletion of *ryhB* prevents APEC from multiplying in the bloodstream and causing bacteremia, and then causing meningitis. Of course, RyhB contributes to the adhesion to BMECs, which may also be an important reason for meningitis. Moreover, the critical virulence factors that RyhB regulates remains unclear. Further studies are needed to determine the virulence factors related to meningitis regulated by RyhB. In addition, although the deletion of *ryhB* resulted in the expression changes of more than 200 genes, it does not mean that these 200 genes are directly regulated by RyhB. It is possible that the direct targets of RyhB, especially the targets with a regulatory role, regulate the expressions of their targets. The direct targets of RyhB need to be further screened and validated.

## 4. Materials and Methods

### 4.1. Bacterial Strains, Plasmids and Growth Conditions

The bacteria and plasmids used in this study are listed in Table 1. The APEC XM strain (O2:K1) was donated by Dr. Chengping Lu, Nanjing Agricultural University. APEC XMΔ*ryhB* and APEC XMΔ*ryhB*/p*ryhB* constructed in this study were derived from APEC XM. All bacteria were cultured in Luria-Bertani (LB) broth or on LB plates at 37 °C with agitation at 180 rpm. The mutants containing the temperature-sensitive plasmid pCP20 or pKD46 were grown in LB containing ampicillin (Amp, 100 µg/mL) (Sangon Biotech, Shanghai, China) or chloramphenicol (Cm, 34 µg/mL) (Sangon Biotech) when appropriate at 30 °C. For the determination of biofilm formation, APEC XM, APEC XMΔ*ryhB* and APEC XMΔ*ryhB*/p*ryhB* were statically cultured in biofilm-inducing medium (Oxoid, Altrincham, Cheshire, UK) at 37 °C [58]. Plasmids pKD3, pKD46 and pCP20 were used for construction of the deletion mutant. Plasmid pBR322 was used for constructing the complemented mutant.

### 4.2. Construction of the ryhB Deletion Mutant and the Complemented Strain

The *ryhB* deletion mutant was constructed as previously described [59,60]. The primers used in this study are listed in Table S3. Primers containing a 50-bp homologous region extension from the 5′ and 3′ of the *ryhB* sequence were used to amplify the chloramphenicol (Cm) cassette from plasmid pKD3. Allelic replacement of *ryhB* by the Cm cassette and the excision of the Cm cassette were verified by PCR and DNA sequencing. The complemented strain was constructed by transferring the recombined plasmid pBR-*ryhB* to the *ryhB* deletion mutant and verified by PCR.

### 4.3. Survival of APEC in Duck Serum

The serum bactericidal assay was performed as previously described with modification [10,54]. The whole blood duck serum was prepared from 8-week-old pathogen-free ducks. APEC XM, APEC XMΔ*ryhB* and APEC XMΔ*ryhB*/p*ryhB* were cultured in LB medium to the exponential phase. Then, the bacteria were centrifuged, washed twice with PBS to remove the LB medium, resuspended in duck serum with a 1:20 dilution of the original bacteria amount and incubated at 37 °C under low oxygen (2.5%) conditions for 0.5 h, 1 h and 2 h. The survival rate of bacteria at different time points was determined by calculating the ratio of the bacteria number at the above time points to the number at 0 h for each strain.

### 4.4. Adhesion Assay

bEnd. 3 cells were cultured in Dulbecco’s minimal Eagle medium (DMEM) (Gibco, Carlsbad, CA, USA) containing glutamine (Gibco) and 12% fetal bovine serum at 37 °C in 5% CO_2_ atmosphere.

The adhesion assay was performed as previously described [54,60]. APEC XM, APEC XMΔ*ryhB* and APEC XMΔ*ryhB*/p*ryhB* were cultured in LB medium to the mid-log phase. Then, 10^5^ monolayer cells/well were infected with the bacteria at a multiplicity of infection (MOI) of 100 in a 96-well plate for 2 h. Loose adherent bacteria were washed three times with PBS. Cells were lysed with 0.5% Triton X-100 (Solarbio, Beijing, China) for 30 min. The lysates were serially diluted and plated onto LB agar plates to count the adherent bacteria.

### 4.5. Determination of Biofilm Formation

The biofilm formation assay was carried out as previously described with modification [61]. APEC XM, APEC XMΔ*ryhB* and APEC XMΔ*ryhB*/p*ryhB* were cultured in LB liquid medium to OD_600_ = 1. For qualitative assay of biofilm formation, the above activated bacteria were transferred to the biofilm induction medium with a ratio of 1:100, and incubated at 30 °C for 36 h. Then, the cultures were discarded. The tubes were washed with distilled water three times to remove unattached bacteria. Each tube was stained with 0.1% crystal violet solution, incubated at room temperature for 5 min and washed three times. Then, the formation of a violet ring was observed. For the quantitative assay, the same protocol of staining with crystal violet was performed in a 96-well plate. Then, the crystal violet in the well was dissolved with 95% ethanol for 5 min. The absorbance of OD_600_ was measured using a multifunctional enzyme marker (BioTek, Winooski, VT, USA).

### 4.6. qRT- PCR

qRT-PCR was carried out as described by Chen et al. [39,54]. All of the primers used to amplify *ryhB*, 10 DEGs from RNA-seq data and cytokine genes *IL-1β*, *IL-6* and *TNF-α* are shown in Table S4. Total RNA was extracted using TRIzol reagent (Invitrogen, Carlsbad, CA, USA) from bacteria cultured in duck serum or the brain tissue of mice. cDNA was synthesized by using a PrimeScript RRT reagent kit with gDNA Eraser (Takara, Tokyo, Japan). qRT-PCR was performed on an ABI7500 instrument (Applied Biosystems, Carlsbad, CA, USA) using SYBR Premix Ex Taq II (Takara). The relative mRNA expression of each gene was evaluated using the 2^−ΔΔCt^ method. g*apA* and *GAPDH* were used as the endogenous reference genes to detect the expression of DEGs and cytokines, respectively. Assays were performed in triplicate.

### 4.7. RNA-Seq and Data Analysis

APEC XM and APEC XMΔ*ryhB* were grown in LB to the mid log phase, collected by centrifugation, washed in PBS, resuspended in duck serum and incubated for 2 h. Samples of each strain were prepared with three biological repeats. For transcriptome sequencing, total RNA of the above bacterial samples was extracted using TRIzol reagent (Invitrogen) with DNase digestion (Takara). Ribosomal RNA was removed using an Epicentre Ribo-zeroTM rRNA Removal Kit (Epicentre, Madison, WI, USA), and rRNA free residue was cleaned by ethanol precipitation. RNA integrity was evaluated using the RNA Nano 6000 Assay Kit of the Agilent Bioanalyzer 2100 system (Agilent Technologies, Santa Clara, CA, USA). Sequencing libraries were prepared by the NEBNext^®^ UltraTM Directional RNA Library Prep Kit for Illumina^®^ (NEB, Ipswich, MA, USA) following the manufacturer’s recommendations. The library quality was assessed on the Agilent Bioanalyzer 2100 system. The clustering of the index-coded samples was performed on a cBot Cluster Generation System using Novaseq 6000 PE Cluster Kit (Illumina, San Diego, CA, USA). Then the library preparations were sequenced on an Illumina Novaseq 6000 platform (Illumina) to generate 150 bp paired-end reads. The RNA-seq clean reads were aligned to the genome of *E. coli* strain ExPEC XM from NCBI (https://www.ncbi.nlm.nih.gov/assembly/GCF_002844685.1/#/def (accessed on 19 December 2017)). Gene expression was quantified as reads per kilobase of coding sequence per million reads (RPKM). Based on a comparison of comparing the transcriptome data of APEC XM and APEC XMΔ*ryhB* challenged with duck serum, genes with an adjusted |log2(fold change) | ≥1 and false discovery rate (FDR) < 0.01 were used as a standard to screen differentially expressed genes (DEGs). The Benjamini–Hochberg correction method was used to correct the *p*-value obtained from the original hypothesis test. The GO and KEGG databases were used for the functional annotation and enrichment of DEGs.

### 4.8. Establishment of Mouse Meningitis Model Infected with APEC

The mouse meningitis model was established as previously described [62]. APEC XM, APEC XMΔ*ryhB* and APEC XMΔ*ryhB*/p*ryhB* were grown in LB to the mid log phase, collected by centrifugation and diluted in PBS for mice inoculation. In the animal experiment, 4-week-old ICR mice were provided by the Comparative Medicine Center of Yangzhou University (License number: SCXK (Su) 2017–0007). All mice had free access to food and water under a 12:12 h (L:D) photoperiod. Forty mice were randomly separated into a negative control group and three infection groups (infected with APEC XM, APEC XMΔ*ryhB* or APEC XMΔ*ryhB*/p*ryhB*). Then, 1 × 10^7^ CFU bacteria in 100 µL PBS were inoculated into each mouse by intraperitoneal injection. The mice in the negative control group received 100 µL PBS with the same method. The status and clinical symptoms of the mice were monitored for 12 h. The health status of the mice was evaluated by a clinical score as previously described (0, no apparent behavioral abnormality; 1, moderate lethargy; 2, severe lethargy; 3, unable to walk; 4, dead) [63]. At 12 h post infection, the mice were euthanized by manual cervical dislocation within approximately 10 s for ethical reasons. Blood, brains, spleen and lungs were immediately collected after euthanasia. None of the mice spontaneously died.

The animal experiments were carried out according to the National Institute of Health guidelines for the ethical use of animals in China. All animal protocols were approved by the Animal Care and Ethics Committee of Yangzhou University (code No. 202102003).

### 4.9. Determination of Bacterial Loadings in the Tissues of Mice

The bacterial loadings in the tissues were determined as previously described [62]. After euthanasia, the blood, right hemisphere of the brain, lung and spleen were aseptically collected and homogenized with sterile pre-cooled PBS. The homogenates were serially diluted (tenfold dilution) and plated on MacConkey plates. The bacterial loadings were calculated as the CFU per gram of tissues or per microliter of blood.

### 4.10. Detection of Lesions of the Brain by MRI Scanning and Histological Analysis

To determine brain lesions, MRI scanning of the brain [64,65] and histological analysis [65] were performed as previously described. The mice were anaesthetized by 2% isoflurane inhalation and then maintained with 1.5% isoflurane 12 h post infection. The brain was scanned using T1WI and enhanced T1WI on a 7.0-T MRI scanner (Bruker Corporation, BRUKER BIOSPEC 70/30, Billerica, MA, USA). For histological analysis, the mice were euthanized 12 h post infection. The brains were collected, fixed with 4% paraformaldehyde and embedded with paraffin. The 3–4 µm sections were prepared by an automated microtome (Leica RM2255, Wetzlar, Germany), stained with hematoxylin and eosin (H&E) and observed by a microscope (Nikon, Eclipse 80i, Tokyo, Japan).

### 4.11. Brain Permeability of Evans Blue

Brain permeability of EB can be used to evaluate the integrity of the BBB. The assay of the EB amount was carried out as previously described [54,66]. Each mouse was injected with 100 µL 2% EB solution into the caudal vein 30 min before euthanasia. The amount of EB that penetrated the brain was calculated by measuring the absorbance at 630 nm using a spectrophotometer.

### 4.12. Detection of Tight Junction Protein Expression by Western Blot and Immunohistochemistry

The expressions of tight junction proteins ZO-1, claudin-5 and occludin were examined by Western blot and immunohistochemistry as previously described [62]. The sections were prepared as mentioned in the “histological analysis” section. Then, they were incubated with primary antibodies ZO-1, occludin and claudin-5 (Invitrogen) and the horseradish peroxidase (HRP) labeling secondary antibody (Boster Biological Technology, Shanghai, China), counterstained with hematoxylin, dehydrated and observed with a microscope (Leica TCS SP8, Wetzlar, Germany). Images were analyzed using Image J software (Image-Pro Plus 6.0, MediaCybernetics, Rockville, MD, USA).

### 4.13. Statistical Analysis

Data were analyzed with SPSS 17.0 software (SPSS, Chicago, IL, USA) using one-way ANOVA for multiple comparisons and represented as the mean ± SEM based on triplicate independent experiments. For significance difference analysis, *p*-value < 0.05 was considered to be significant.

## 5. Conclusions

RyhB contributes to the biofilm formation, adhesion to b. End 3 cells and the serum resistance ability of APEC. RyhB in APEC regulates multiple virulence-related genes, such as fimbriae synthesis genes and iron metabolism-related genes, when resisting the duck serum. RyhB plays an important role in meningitis development in mice infected with APEC.

## Figures and Tables

**Table 1:** Bacterial strains and plasmids used in this study.

Strain or Plasmid	Characteristic or Function	Source and Reference
APEC XM	Virulent strain of APEC, isolated from the brain of duck	Donated by Dr. Chengping Lu
APEC XMΔ*ryhB*	Deletion mutant of *ryhB* with APEC XM background	This study
APEC XMΔ*ryhB*/p*ryhB*	APEC XM Δ*ryhB* carrying the vector pBR-*ryhB*, Amp ^r^	This study
pKD46	Amp ^r^, λ red recombinase expression	[59]
pKD3	Cm ^r^; Cm cassette template	[59]
pCP20	Amp^r^, Cm ^r^, Flp recombinase expression	[59]
pBR-*ryhB*	Amp ^r^, pBR322 carrying the full *ryhB* gene sequence	This study

## Data Availability

The datasets generated and/or analyzed during the current study are not publicly available due to the project is not finished yet but are available from the corresponding author on reasonable request.

## Supplementary Materials

The supporting information can be downloaded at: https://www.mdpi.com/article/10.3390/ijms232415532/s1.

## References

[1] Kasuya K., Shimokubo N., Kosuge C., Takayama K., Yoshida E., Osaka H. (2016). Three cases of *Escherichia coli* meningitis in chicks imported to Japan. Avian Dis..

[2] Tivendale K.A., Logue C.M., Kariyawasam S., Jordan D., Hussein A., Li G.W., Wannemuehler Y., Nolan L.K. (2010). Avian-pathogenic *Escherichia coli* strains are similar to neonatal meningitis *E. coli* strains and are able to cause meningitis in the rat model of human disease. Infect. Immun..

[3] Moulin-Schouleur M., Schouler C., Tailliez P., Kao M.R., Brée A., Germon P., Oswald E., Mainil J., Blanco M., Blanco J. (2006). Common virulence factors and genetic relationships between O18:K1:H7 *Escherichia coli* isolates of human and avian origin. J. Clin. Microbiol..

[4] Ewers C., Li G., Wilking H., Kiessling S., Alt K., Antáo E.M., Laturnus C., Diehl I., Glodde S., Homeier T. (2007). Avian pathogenic, uropathogenic, and newborn meningitis-causing *Escherichia coli*: How closely related are they?. Int. J. Med. Microbiol..

[5] Johnson T.J., Kariyawasam S., Wannemuehler Y., Mangiamele P., Johnson S.J., Doetkott C., Skyberg J.A., Lynne A.M., Johnson J.R., Nolan L.K. (2007). The genome sequence of avian pathogenic *Escherichia coli* strain O1:K1:H7 shares strong similarities with human extraintestinal pathogenic *E. coli* genomes. J. Bacteriol..

[6] Krishnan S., Chang A.C., Hodges J., Couraud P.O., Romero I.A., Weksler B., Nicholson B.A., Nolan L.K., Prasadarao N.V. (2015). Serotype O18 avian pathogenic and neonatal meningitis *Escherichia coli* strains employ similar pathogenic strategies for the onset of meningitis. Virulence.

[7] Kim K.S. (2020). Investigating Bacterial Penetration of the Blood Brain Barrier for the Pathogenesis, Prevention, and Therapy of Bacterial Meningitis. ACS Infect. Dis..

[8] Kim K.S. (2003). Pathogenesis of bacterial meningitis: From bacteraemia to neuronal injury. Nat. Rev. Neurosci..

[9] Ma J., Bao Y., Sun M., Dong W., Pan Z., Zhang W., Lu C., Yao H. (2014). Two Functional Type VI Secretion Systems in Avian Pathogenic *Escherichia coli* Are Involved in Different Pathogenic Pathways. Infect. Immun..

[10] Ma J., An C., Jiang F., Yao H., Logue C., Nolan L.K., Li G. (2018). Extraintestinal pathogenic *Escherichia coli* increase extracytoplasmic polysaccharide biosynthesis for serum resistance in response to bloodstream signals. Mol. Microbiol..

[11] Hejair H.M.A., Zhu Y., Ma J., Zhang Y., Pan Z., Zhang W., Yao H. (2017). Functional role of *ompF* and *ompC* porins in pathogenesis of avian pathogenic *Escherichia coli*. Microb. Pathog..

[12] Sun H., Song Y., Chen F., Zhou C., Liu P., Fan Y., Zheng Y., Wan X., Feng L. (2020). An ArcA-Modulated Small RNA in Pathogenic *Escherichia coli* K1. Front. Microbiol..

[13] Mahendran G., Jayasinghe O.T., Thavakumaran D., Arachchilage G.M., Silva G.N. (2022). Key players in regulatory RNA realm of bacteria. Biochem. Biophys. Rep..

[14] Jørgensen M.G., Pettersen J.S., Kallipolitis B.H. (2020). sRNA-mediated control in bacteria: An increasing diversity of regulatory mechanisms. Biochim. Biophys. Acta Gene Regul. Mech..

[15] Felden B., Augagneur Y. (2021). Diversity and Versatility in Small RNA-Mediated Regulation in Bacterial Pathogens. Front. Microbiol..

[16] Dutta T., Srivastava S. (2018). Small RNA-mediated regulation in bacteria: A growing palette of diverse mechanisms. Gene.

[17] Wassarman K.M., Repoila F., Rosenow C., Storz G., Gottesman S. (2001). Identification of novel small RNAs using comparative genomics and microarrays. Genes Dev..

[18] Kim J.N., Kwon Y.M. (2013). Genetic and phenotypic characterization of the RyhB regulon in *Salmonella* Typhimurium. Microbiol. Res..

[19] Leclerc J.M., Dozois C.M., Daigle F. (2013). Role of the *Salmonella enterica* serovar Typhi Fur regulator and small RNAs RfrA and RfrB in iron homeostasis and interaction with host cells. Microbiology.

[20] Mey A.R., Craig S.A., Payne S.M. (2005). Characterization of *Vibrio cholerae* RyhB: The RyhB regulon and role of *ryhB* in biofilm formation. Infect. Immun..

[21] Deng Z., Meng X., Su S., Liu Z., Ji X., Zhang Y., Zhao X., Wang X., Yang R., Han Y. (2012). Two sRNA RyhB homologs from *Yersinia pestis* biovar microtus expressed in vivo have differential Hfq-dependent stability. Res. Microbiol..

[22] Huang S.H., Wang C.K., Peng H.L., Wu C.C., Chen Y.T., Hong Y.M., Lin C.T. (2012). Role of the small RNA RyhB in the Fur regulon in mediating the capsular polysaccharide biosynthesis and iron acquisition systems in *Klebsiella pneumoniae*. BMC Microbiol..

[23] Murphy E.R., Payne S.M. (2007). RyhB, an iron-responsive small RNA molecule, regulates *Shigella dysenteriae* virulence. Infect. Immun..

[24] Massé E., Gottesman S. (2002). A small RNA regulates the expression of genes involved in iron metabolism in *Escherichia coli*. Proc. Natl. Acad. Sci. USA.

[25] Kim J.N. (2016). Roles of two RyhB paralogs in the physiology of *Salmonella enterica*. Microbiol. Res..

[26] Chareyre S., Mandin P. (2018). Bacterial Iron Homeostasis Regulation by sRNAs. Microbiol. Spectr..

[27] Calderón P.F., Morales E.H., Acuña L.G., Fuentes D.N., Gil F., Porwollik S., McClelland M., Saavedra C.P., Calderón I.L. (2014). The small RNA RyhB homologs from *Salmonella typhimurium* participate in the response to S-nitrosoglutathione-induced stress. Biochem. Biophys. Res. Commun..

[28] Oglesby A.G., Murphy E.R., Iyer V.R., Payne S.M. (2005). Fur regulates acid resistance in *Shigella flexneri* via RyhB and ydeP. Mol. Microbiol..

[29] Calderón I.L., Morales E.H., Collao B., Calderón P.F., Chahuán C.A., Acuña L.G., Gil F., Saavedra C.P. (2014). Role of *Salmonella* Typhimurium small RNAs RyhB-1 and RyhB-2 in the oxidative stress response. Res. Microbiol..

[30] Kim J.N., Kwon Y.M. (2013). Identification of target transcripts regulated by small RNA RyhB homologs in *Salmonella*: RyhB-2 regulates motility phenotype. Microbiol. Res..

[31] Peñaloza D., Acuña L.G., Barros M.J., Núñez P., Montt F., Gil F., Fuentes J.A., Calderón I.L. (2021). The Small RNA RyhB Homologs from *Salmonella* Typhimurium Restrain the Intracellular Growth and Modulate the SPI-1 Gene Expression within RAW264.7 Macrophages. Microorganisms.

[32] Porcheron G., Habib R., Houle S., Caza M., Lépine F., Daigle F., Massé E., Dozois C.M. (2014). The small RNA RyhB contributes to siderophore production and virulence of uropathogenic *Escherichia coli*. Infect. Immun..

[33] Padilla-Vaca F., Mondragón-Jaimes V., Franco B. (2017). General Aspects of Two-Component Regulatory Circuits in Bacteria: Domains, Signals and Roles. Curr. Protein Pept. Sci..

[34] Abisado R.G., Benomar S., Klaus J.R., Dandekar A.A., Chandler J.R. (2018). Bacterial Quorum Sensing and Microbial Community Interactions. mBio.

[35] Kim K.S. (2016). Human Meningitis-Associated *Escherichia coli*. EcoSal Plus.

[36] Papenfort K., Vogel J. (2010). Regulatory RNA in bacterial pathogens. Cell Host Microbe.

[37] Mellata M., Dho-Moulin M., Dozois C.M., Curtiss R., Brown P.K., Arné P., Brée A., Desautels C., Fairbrother J.M. (2003). Role of virulence factors in resistance of avian pathogenic *Escherichia coli* to serum and in pathogenicity. Infect. Immun..

[38] Oglesby-Sherrouse A.G., Murphy E.R. (2013). Iron-responsive bacterial small RNAs: Variations on a theme. Metallomics.

[39] Chen B., Meng X., Ni J., He M., Chen Y., Xia P., Wang H., Liu S., Zhu G., Meng X. (2021). Positive regulation of Type III secretion effectors and virulence by RyhB paralogs in *Salmonella enterica* serovar Enteritidis. Vet. Res..

[40] Beauchene N.A., Myers K.S., Chung D., Park D.M., Weisnicht A.M., Keleş S., Kiley P.J. (2015). Impact of Anaerobiosis on Expression of the Iron-Responsive Fur and RyhB Regulons. mBio.

[41] Padalon-Brauch G., Hershberg R., Elgrably-Weiss M., Baruch K., Rosenshine I., Margalit H., Altuvia S. (2008). Small RNAs encoded within genetic islands of *Salmonella typhimurium* show host-induced expression and role in virulence. Nucleic Acids Res..

[42] Suerbaum S., Friedrich S., Leying H., Opferkuch W. (1994). Expression of capsular polysaccharide determines serum resistance in *Escherichia coli* K92. Zentralbl. Bakteriol..

[43] Cross A.S., Kim K.S., Wright D.C., Sadoff J.C., Gemski P. (1986). Role of lipopolysaccharide and capsule in the serum resistance of bacteremic strains of *Escherichia coli*. J. Infect. Dis..

[44] Prasadarao N.V., Blom A.M., Villoutreix B.O., Linsangan L.C. (2002). A novel interaction of outer membrane protein A with C4b binding protein mediates serum resistance of *Escherichia coli* K1. J. Immunol..

[45] Yair Y., Michaux C., Biran D., Bernhard J., Vogel J., Barquist L., Ron E.Z. (2022). Cellular RNA Targets of Cold Shock Proteins CspC and CspE and Their Importance for Serum Resistance in Septicemic *Escherichia coli*. mSystems.

[46] Huja S., Oren Y., Biran D., Meyer S., Dobrindt U., Bernhard J., Becher D., Hecker M., Sorek R., Ron E.Z. (2014). Fur is the master regulator of the extraintestinal pathogenic *Escherichia coli* response to serum. mBio.

[47] Wang S., Niu C., Shi Z., Xia Y., Yaqoob M., Dai J., Lu C. (2011). Effects of ibeA deletion on virulence and biofilm formation of avian pathogenic *Escherichia coli*. Infect. Immun..

[48] Zhuge X., Wang S., Fan H., Pan Z., Ren J., Yi L., Meng Q., Yang X., Lu C., Dai J. (2013). Characterization and functional analysis of AatB, a novel autotransporter adhesin and virulence factor of avian pathogenic *Escherichia coli*. Infect. Immun..

[49] Palmela C., Chevarin C., Xu Z., Torres J., Sevrin G., Hirten R., Barnich N., Ng S.C., Colombel J.F. (2018). Adherent-invasive *Escherichia coli* in inflammatory bowel disease. Gut.

[50] Behzadi P. (2020). Classical chaperone-usher (CU) adhesive fimbriome: Uropathogenic *Escherichia coli* (UPEC) and urinary tract infections (UTIs). Folia Microbiol. (Praha).

[51] Duan Q., Zhou M., Zhu X., Yang Y., Zhu J., Bao W., Wu S., Ruan X., Zhang W., Zhu G. (2013). Flagella from F18+*Escherichia coli* play a role in adhesion to pig epithelial cell lines. Microb. Pathog..

[52] Del Pozo J.L. (2018). Biofilm-related disease. Expert Rev. Anti-Infect. Ther..

[53] Xiao G., Tang H., Zhang S., Ren H., Dai J., Lai L., Lu C., Yao H., Fan H., Wu Z. (2017). *Streptococcus suis* small RNA rss04 contributes to the induction of meningitis by regulating capsule synthesis and by inducing biofilm formation in a mouse infection model. Vet. Microbiol..

[54] Wang P., Zhang J., Chen Y., Zhong H., Wang H., Li J., Zhu G., Xia P., Cui L., Li J. (2021). Colibactin in avian pathogenic *Escherichia coli* contributes to the development of meningitis in a mouse model. Virulence.

[55] Kim K.S. (2008). Mechanisms of microbial traversal of the blood-brain barrier. Nat. Rev. Microbiol..

[56] Furuse M. (2010). Molecular basis of the core structure of tight junctions. Cold Spring Harb. Perspect. Biol..

[57] Coureuil M., Lécuyer H., Bourdoulous S., Nassif X. (2017). A journey into the brain: Insight into how bacterial pathogens cross blood-brain barriers. Nat. Rev. Microbiol..

[58] Zhong H., Wang P., Chen Y., Wang H., Li J., Li J., Zhu G., Cui L., Meng X. (2021). ClpV1 in avian pathogenic *Escherichia coli* is a crucial virulence factor contributing to meningitis in a mouse model in vivo. Vet. Microbiol..

[59] Datsenko K.A., Wanner B.L. (2000). One-step inactivation of chromosomal genes in *Escherichia coli* K-12 using PCR products. Proc. Natl. Acad. Sci. USA.

[60] Meng X., Meng X., Wang J., Wang H., Zhu C., Ni J., Zhu G. (2019). Small non-coding RNA STnc640 regulates expression of *fimA* fimbrial gene and virulence of *Salmonella enterica* serovar Enteritidis. BMC Vet. Res..

[61] Hossain M.M., Tsuyumu S. (2006). Flagella-mediated motility is required for biofilm formation by *Erwinia carotovora* subsp. carotovora. J. Gen. Plant. Pathol..

[62] Wang P., Zhang J., Chen Y., Zhong H., Wang H., Li J., Zhu G., Xia P., Cui L., Li J. (2021). ClbG in avian pathogenic *Escherichia coli* contributes to meningitis development in a mouse model. Toxins.

[63] Gerber J., Raivich G., Wellmer A., Noeske C., Kunst T., Werner A., Brück W., Nau R. (2001). A mouse model of *Streptococcus pneumoniae* meningitis mimicking several features of human disease. Acta Neuropathol..

[64] Shyu L.Y., Tsai H.H., Lin D.P., Chang H.H., Tyan Y.S., Weng J.C. (2014). An 8-week brain MRI follow-up analysis of rat eosinophilic meningitis caused by *Angiostrongylus cantonensis* infection. Zoonoses Public Health.

[65] Meng X., Chen Y., Wang P., Xia P., Wang J., He M., Zhu C., Wang H., Zhu G. (2022). Phosphopantetheinyl transferase ClbA contributes to the virulence of avian pathogenic *Escherichia coli* in meningitis infection of mice. PLoS ONE.

[66] Manaenko A., Chen H., Kammer J., Zhang J.H., Tang J. (2011). Comparison Evans Blue injection routes: Intravenous versus intraperitoneal, for measurement of blood-brain barrier in a mice hemorrhage model. J. Neurosci. Methods.

