# Supplementary material for: RyhB in Avian Pathogenic Escherichia coli Regulates the Expression of Virulence-Related Genes and Contributes to Meningitis Development in a Mouse Model
Source: Int J Mol Sci. 2022 Dec 8;23(24):15532. doi: 10.3390/ijms232415532 (PMC9778962; doi:10.3390/ijms232415532)
Supplement: Supplementary file 1 [file ijms-23-15532-s001.zip › ijms-2035260-supplementary.pdf]

Supplementary materials

Figure S1

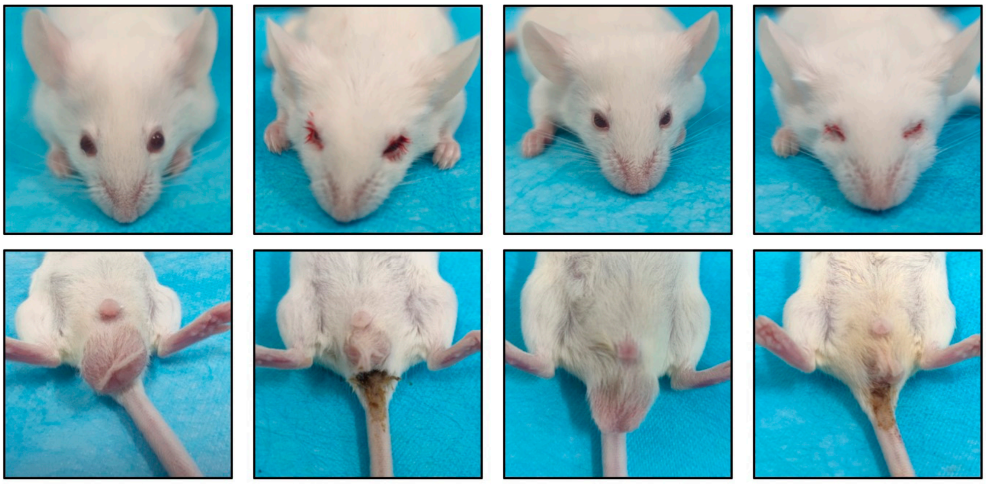

Figure S1. Clinical symptoms of mice after 12h infection.

Table S1 Details of 10 DEGs used for verification

Gene name	Length	Product	log2(FC) WT vs APEC XM△*ryh*B
fimA1	555	Type-1 fimbrial protein	4.311447
fimF	540	Fimbrial protein	3.526543952
fimI	531	Fimbrin-like protein FimI	4.029273495
sodB	582	Inorganicion transport and metabolism	2.107802072
chuS	1029	Inorganic ion transport and metabolism	1.680109428
neuB	1041	Cellwall/membrane/envelope biogenesis	-7.870757016
feoB	2322	Ferrous iron transport protein B	-1.153400871
cdtA	714	Cytolethal distending toxin A/C domain	-3.678002453
feoA	228	Fe(2+) transport protein A	-2.54383029
fimA2	564	Fimbrial protein	-4.952563136

Table S2 The details of DEGs enriched in KEGG mentioned in the manuscript.

Gene name	Length	Product	APEC vs APEC XM△*ryh*B			
			log2(FC)	pValue	FDR	regulated
**Two-component system Pathway**						
mdtA	1248	Multidrug resistance protein	-1.2359	7.39E-07	1.62E-05	down
mdtC	3078	Multidrug resistance protein	-1.5260	6.25E-10	2.19E-08	down
mdtB	3123	Multidrug resistance protein	-1.4235	1.45E-07	3.61E-06	down
dcuB	1340	Anaerobic C4-dicarboxylate transporter	1.7290	2.64E-11	1.05E-09	up
iroN	2178	Ferric enterobactin receptor	-3.2883	1.33E-50	2.19E-48	down
arnB	1140	Cell/membrane/envelope biogenesis	-1.2380	1.99E-06	4.14E-05	down
flhD	351	Flagellar transcriptional regulator	1.0392	1.79E-05	0.000311	up
flhC	579	Flagellar transcriptional regulator	0.9862	0.00192	0.017702	normal
fdnG	3048	Formate dehydrogenase-N subunit alpha	1.7628	1.52E-18	9.01E-17	up
fdnH	885	Formate iron-sulfur subunit	1.1741	3.21E-07	7.49E-06	up
frdD	360	Fumarate reductase subunit D	1.0603	8.70E-06	0.000161	up
frdC	396	Fumarate reductase subunit C	1.1688	2.71E-06	5.59E-05	up
uhpA	591	Transcriptional regulatory protein	1.5582	2.55E-08	7.09E-07	up
uvrY	657	BarA-associated response regulator	-1.2521	4.03E-13	1.79E-11	down
evgS	3594	acid-sensing system histidine kinase	-2.5098	9.33E-20	5.90E-18	down
atoS	1827	Signal transduction histidine-protein kinase	-1.1007	1.20E-08	3.55E-07	down
dgcT	1368	Probable diguanylate cyclase DgcT	-1.3296	5.46E-06	0.000105	down
grpP	1263	Streptococcus mutans serotype C	-1.0089	4.05E-06	8.07E-05	down

Quorum sensing Pathway						
trpE	1563	Anthranilate synthase component 1	1.8502	1.19E-09	3.91E-08	up
flhC	579	Flagellar transcriptional regulator	0.9862	0.001927	0.017703	normal
flhD	351	Flagellar transcriptional regulator	1.0392	1.79E-05	0.000312	up
rcsA	624	Transcriptional regulatory protein	1.3705	3.41E-05	0.000562	up
ygjI	1434	Inner membrane transporter YgjI	1.2530	0.001718	0.016072	normal
The capsules biosynthesis DEGs						
kpsE	1149	Capsule biosynthesis protein	-3.7873	2.54E-32	2.50E-30	down
kpsF	984	Arabinose 5-phosphate isomerase	-6.3868	1.29E-68	2.59E-66	down
kpsS	1206	Capsule polysaccharide export protein	-3.9773	3.79E-27	3.02E-25	down
kpsD	1677	Polysialic acid transport protein	-3.5256	1.56E-26	1.22E-24	down
The serum resistance DEGs						
neuB	1041	N,N'-diacetyllegionaminic acid synthase	-7.6954	0	0	down
neuC	1176	Polysialic acid biosynthesis protein P7	-6.7473	2.42E-193	1.60E-190	down
neuD	624	Bacterial transferase hexapeptide	-8.1722	1.53E-268	2.36E-265	down
Type I fimbria encoded DEGs						
fimA	564	Type-1 fimbrial protein, A chain	-4.9525	3.02E-293	1.95E-290	down
fimH	903	Type 1 fimbrin D-mannose specific adhesin	2.9489	3.80E-41	3.24E-39	up
fimF	531	type 1 fimbria minor subunit	3.5265	6.79E-65	9.88E-63	up
fimA2	555	Type 1 fimbriae major subunit	4.3114	1.32E-130	3.30E-128	up
fimG	504	Minor fimbrial subunit, polypeptide	3.6589	4.57E-61	5.89E-59	up
fimC	726	Chaperone protein	4.0621	4.60E-73	9.66E-71	up

LPS biosynthesis DEGs						
arnB	1140	UDP-4-amino-4-deoxy-L-arabinose aminotransferase	-1.2380	1.99E-06	4.14E-05	down
arnC	969	Cell /membrane/envelope biogenesis	-1.2418	8.72E-05	0.00123	down
Biofilm formation regulator DEGs						
ybaJ	375	Biofilm formation regulator YbaJ	-1.2317	8.22E-11	3.12E-09	down
pgaD	414	Biofilm PGA synthesis protein PgaD	-2.8492	3.26E-25	2.43E-23	down

Table S3 Primers used for mutants construction.

Primer	Sequences (5′-3′)	Description
RyhB-DP-F	CGCGTGTTTCTGCGTGGCGTATTACTATCTCACTCATTCCTTTGTCCTCTTTGGGGTGTGTAGGCTGGAGCTGCTTCG	Construction of isogenic *ryhB* mutant
RyhB-DP-R	TAAGGTGGTTATTTACACCTTAGCGCAAAGCGGACGTGGTTCCTACTGGAGTCATATGAATATCCTCCTTAG	Construction of isogenic *ryhB* mutant
RyhB-IP-F	GCACCTGTAGCGTGTTGTA	Confirmed isogenic *ryhB* mutant
RyhB-IP-R	TGCCGAAGCCAATAAAGGC	Confirmed isogenic *ryhB* mutant
RyhB-pBR-F	TAACGCAGTCAGGCACCGTGTATCTCACTCATTCCTTTGTCCTCTTTG	Construction of pBR-*ryhB*
RyhB-pBR-R	GTGAATCCGTTAGCGAGGTGCCCAATAAAGGCGCAGTTAATGACCA	Construction of pBR-*ryhB*

Table S4 The sequence of primers for qRT-PCR

Primer	Sequences (5′-3′)
ryhB-F	CGATCAGGAAGACCCTCGC
ryhB-R	CACCCGGCTGGCTAAGTAAT
fimA-F	TTGTTCTGTCGGCTCTGTCC
fimA -R	GTCCCACCATTTACCGTCGT
fimI -F	GGTTTCATGCGGTAGGGGAA

fimI -R	TTCACTCACCACCGTGCTAC
fimF -F	GATCTGATGGAAAACGCGGC
fimF -R	ACCACAGGGTGACAGCAAAA
sodB -F	CTTTTGGCAGCTTTGCCGAT
sodB -R	GGTTTCATGCGGTAGGGGAA
chuS -F	ACTCTTGCTCGACCACACTG
chuS -R	TCACCGATGAGAATACGCCG
neuB -F	GGAGTTTTCGACCGGCCTTA
neuB -R	AATATCCGGGCGCTGCAATA
feoB -F	ATCTGTATCTGACGCAGCCG
feoB -R	TGTACATATGACGCGTCGGG
feoA -F	GACTACCCTGGTTCAGTGCC
feoA -R	AGGTGAAAACGACGCGGTAT
cdtA-F	GCGCTGCTTACTATTTGGGC
cdtA-R	CCACAACCTTCCAGTTCCGA
fimA2-F	AGGCGAGAAGAACGAACCTG
fimA2-R	GTAAATGCCGCTTGTGCGAT
gapA-F	CGTTAAAGGCGCTAACTTCG
gapA-R	ACGGTGGTCATCAGACCTTC
TNF-α-F	ACTGAACTTCGGGGTGATCG
TNF-α-R	TGATCTGAGTGTGAGGGTCTGG
IL-1β-F	ATGAAAGACGGCACACCCAC
IL-1β-R	GCTTGTGCTCTGCTTGTGAG
IL-6-F	TGCAAGAGACTTCCATCCAGT
IL-6-R	GTGAAGTAGGGAAGGCCG
GAPDH-F	AACGGGAAGCCCATCACCATC
GAPDH-R	AAGACACCAGTAGACTCCACGA
